# Clinical and immunological features of platelet transfusion refractoriness in young patients with de novo acute myeloid leukemia

**DOI:** 10.1002/cam4.3140

**Published:** 2020-05-18

**Authors:** Xuelian Hu, Haodong Cai, Lu Zheng, Yi Luo, Jing Zhou, Yan Hui, Zhenyu Dai, Haolong Lin, Dengju Li, Yi Xiao, Liang Huang, Jianfeng Zhou

**Affiliations:** ^1^ Department of Hematology Tongji Hospital Tongji Medical College Huazhong University of Science and Technology Wuhan Hubei China; ^2^ Lishui City People's Hospital Zhejiang China

**Keywords:** core binding factor acute myeloid leukemia, human leukocyte antigen, platelet transfusion refractoriness, risk factor

## Abstract

Platelet transfusion is important in the prevention and treatment of bleeding in patients with acute myeloid leukemia (AML) after receiving intensive chemotherapy. However, platelet transfusion refractoriness (PTR) is an intractable clinical issue occurred in these patients. And its clinical and immunological features remain largely unknown. The potential causes and clinical features of PTR were retrospectively analyzed in 560 patients who were diagnosed as de novo AML in Tongji Hospital from June 2012 through June 2018. A high‐throughput antibody screening for the detection of human leukocyte antigen (HLA) and its serotypes was performed in 133 newly diagnosed AML patients. PTR occurred in 11.8% of the de novo AML patients. The median age for patients with PTR was 46 years (range, 15‐70). It frequently manifested in female patients and in patients with splenomegaly, M4 subtype, *c‐Kit* gene mutation, and rearrangements of *RUNX1‐RUNX1T1* or *CBFB‐MYH11*, commonly referred to as core binding factor AML (CBF‐AML). Notably, CBF‐AML was independently associated with the occurrence of PTR. PTR predominantly developed in patients who had CBF‐AML (*P* < .001) and in patients who further had better minimal residual disease (MRD) reduction (≥3‐log) before the second consolidation chemotherapy (*P* = .007). HLA‐I antibodies were detected in the serum of 9.0% of AML patients and markedly enriched in patients with PTR (*P* < .001) and in patients with CBF‐AML (*P* = .018). HLA‐B was the most frequently identified serum epitope in PTR patients. Patients with CBF‐AML had higher tendency to develop HLA‐I antibodies and PTR, which depicted novel features of PTR in AML and might provide insights into its efficient managements.

## Graphical abstract

Platelet transfusion refractoriness (PTR) is an intractable clinical issue with increasing risks of bleeding and mortality in patients with acute myeloid leukemia (AML) after receiving platelet transfusion. In the present manuscript, patients with CBF‐AML and patients who further had better minimal residual disease (MRD) reduction (≥3‐log) after induction chemotherapy had higher tendency to develop HLA‐I antibodies and PTR. Moreover, HLA‐B was the most frequently identified serum epitope in PTR patients.

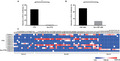

## 1 INTRODUCTION

Platelet transfusion is a common treatment conducted in patients with thrombocytopenia to prevent serious hemorrhage. However, not all patients would achieve the expected platelet count increment after platelet transfusion. Platelet transfusion refractoriness (PTR) is defined as the repeated failures to achieve satisfactory responses to platelet transfusions from random donors, and has become an intractable clinical issue that may increase bleeding risks and health‐care costs.^1^, ^2^ In patients with cancer, the incidence of PTR differs from 4.8% to 54.7%, according to its distinct definitions and study populations.^3^, ^4^, ^5^, ^6^ Alloimmunization to HLA antigens and/or human platelet antigens (HPA) is the most common etiology of PTR.^4^, ^7^, ^8^, ^9^, ^10^ Anti‐HLA and/or HPA antibodies directly react with donor‐derived platelets and can decrease their functions in vivo.^11^ Moreover, nonimmune etiologies, including infection, high fever, antibiotics, antifungal medications, heparin, bleeding, splenomegaly, and multiple pregnancies, have also been involved in PTR.^12^

Previously, PTR had been reported in AML patients, with risks of severe bleeding and mortality, after they had received intensive chemotherapy or hematopoietic stem cell transplantation.^3^, ^10^ However, the underline factors linked with PTR in AML patients are largely unknown. Therefore, to better understand the pathogenesis of PTR in AML, we retrospectively analyzed its clinical and immunologic features in 560 patients who were diagnosed as de novo AML in Tongji Hospital from June 2012 through June 2018.

## 2 METHODS

### 2.1 Definition of platelet transfusion refractoriness

PTR is the repeated failure to achieve the desired level of blood platelets in a patient following platelet transfusions. According to the published definitions, PTR in the present study was defined as a posttransfusion platelet increment (PI) that is less than 5 × 10^9^/L after receiving at least two successive daily platelet transfusions.^2^, ^13^, ^14^

### 2.2 Patients enrollment

De novo AML patients diagnosed in our department were successively enrolled in this study. Patients with secondary or myelodysplastic syndromes/myeloproliferative neoplasms (MDS/ MPN)‐transformed AML were excluded. Clinical and laboratory features of eligible patients were retrospectively reviewed. Risk assessment and risk stratification‐guided treatment were administrated as previously ^15^, ^16^, ^17^ and described in the Supplementary Methods.

This study was approved by the Institutional Review Board of Tongji Hospital, Tongji Medical College, Huazhong University of Science and Technology. Informed consent was obtained from each individual in accordance with the principles expressed in the Declaration of Helsinki.

### 2.3 Detection of anti‐HLA class I antibodies

Whole blood samples were collected from patients with procoagulation tubes when platelet counts were lower than 20 × 10^9^/L after induction chemotherapy. Then serum was separated by centrifugation at 2500 revolutions per minute and frozen at −80℃. The storage time was less than 3 months.

Luminex single antigen beads (SAB) was used in a high‐throughput HLA antibody screening assay (LABScreen Single Antigen HLA Class I, One Lambda) to evaluate the existence of antibodies targeting any donor‐specific HLA class I antigens in the serum, which were the main immunological cause. ^18^, ^19^ This assay was conducted according to the manufacturer's instructions.^20^, ^21^, ^22^ Briefly, 20 μL serum was incubated with 5μL microbeads in a 96‐well V‐bottomed plate in the dark at 25℃ for 30 minutes. After three times of washing, R‐Phycoerythrin‐conjugated goat antihuman IgG antibody was added to the plate for another 30 minutes incubation. The plate was washed for two more times. The single antigen detection beads can identify the following HLA‐I specificities: HLA‐A1, 3, 11, 23‐24, 29‐31, 33‐34, 36, 66, 74, and 80; HLA‐B7, 8, 13, 18, 27, 35, 37‐39, 41, 42, 44‐65, 67, 71‐73, 75‐78, 81, and 82; and HLA‐Cw1, 2, 4‐6, 8‐10, 12, and 14‐18.^23^ Mean fluorescence intensities (MFI) were acquired using a Luminex analyzer. The results were considered to be positive when MFI was above 5000 and strongly positive when MFI was higher than 10 000.^24^, ^25^

### 2.4 Statistical analysis

The patients’ characteristics were described by numbers and frequencies for categorical variables, and by medians and interquartile ranges (IQR) for continuous variables. Differences were analyzed using chi‐square test (or Fisher's exact test in case of small expected numbers) for categorical variables and Student's *t* test for continuous variables. A logistic regression model for multivariate analysis was used to evaluate the risk factors associated with PTR in univariate analysis. *P* values less than .050 (two tailed) were considered to be statistically significant. All statistical analyses were performed using Statistical Package for the Social Sciences (SPSS) version 22.0 (IBM) and GraphPad Prism 6.

## 3 RESULTS

### 3.1 Occurrence of PTR in patients with de novo AML

From June 2012 through June 2018, a total of 560 patients with de novo AML were screened for eligibility. The median age was 45 years (range, 12‐86) with a male‐to‐female ratio of nearly 4:3. After induction chemotherapy, platelet transfusions were given to patients with active hemorrhage or patients with platelet counts less than 20.0 × 10^9^/L. PTR occurred in 66 (11.8%) patients when they received platelet transfusion from random donors. The median time interval between the conduction of chemotherapy and the onset of PTR was 9.0 days (IQR: 6.0‐17.0 days). The median posttransfusion increments with platelets derived from random donors in PTR patients were 0.5 × 10^9^/L (IQR: −3.0‐3.5 × 10^9^/L). Among the 32 PTR patients who were transfused with HLA‐matched platelets, 10 (31.3%) patients showed responses with a median posttransfusion PI of 26.0 × 10^9^/L (IQR: 17.8‐36.3 × 10^9^/L). And the posttransfusion PI of HLA‐matched platelets was significantly higher compared to nonmatched platelet from random donors (9.6 × 10^9^/L vs −0.7 × 10^9^/L, *P *< .001, Figure S1). Other management strategies, including intravenous gamma‐globulin and glucocorticoid, were not effective enough to improve platelet response. Severe bleeding events during induction therapy occurred in 5 PTR patients (7.6%), involving gastrointestinal track (n = 2), cerebrum (n = 1), lung (n = 1), and multiple organs (n = 1).

The median age for patients with PTR was 46 years (range, 15‐70) with a female predominance (male‐to‐female ratio, 2:3, *P* = .007). Patients with splenomegaly were more likely to develop PTR (27.3% vs 14.4%, *P* = .007). However, differences in history of transfusion, pregnancy, or autoimmune diseases, the attack of fever or infection, and the use of antibiotics or liposomal amphotericin B were consistent in patients with or without PTR (Table 1).

**Table 1:** Univariate analysis of PTR in patients with de novo AML

Factors	PTR	Non‐PTR	*P*‐value
Total, n (%)	66 (11.8)	494 (88.2)	
Age (y), median (IQR)	46 (31.75‐54.0)	45.0 (30‐54.25)	.644
Gender, n (%)			
Male	27 (40.9)	289 (58.5)	.007
Female	39 (59.1)	205 (41.5)	
Pregnancy, n (%)			
No	8 (20.5)	31 (15.1)	.400
Yes	31 (79.5)	174 (84.9)	
Transfusion history, n (%)			
No	49 (76.6)	405 (83.9)	.145
Yes	15 (23.4)	78 (16.1)	
NA	2	11	
Autoimmune Diseases, n (%)			
No	63 (95.5)	487 (98.6)	.102
Yes	3 (4.5)	7 (1.4)	
Fever^a^, n (%)			
No	26 (39.4)	227 (46.0)	.315
Yes	40 (60.6)	267 (54.0)	
Infection^a^, n(%)			
No	59 (89.4)	452 (91.5)	.570
Yes	7 (10.6)	42 (8.5)	
Splenomegaly, n (%)			
No	48 (72.7)	423 (85.6)	.007
Yes	18 (27.3)	71 (14.4)	
Use of antibiotics, n (%)			
No	6 (9.1)	32 (6.5)	.433
Yes	60 (90.9)	462 (93.5)	
Use of liposomal amphotericin B, n (%)			
No	61 (92.4)	442 (89.5)	.457
Yes	5 (7.6)	52 (10.5)	

Abbreviations: AML, acute myeloid leukemia; IQR, interquartile ranges; n, observed number of patients within each treatment group; NA, not available; PTR, platelet transfusion refractoriness.

^a^ At diagnose of PTR

### 3.2 Clinical features of PTR in patients with de novo AML

The clinical features of PTR patients were summarized in Table 2. At the disease onset, the white blood cell counts, platelet counts, and the percentages of bone marrow blasts were comparable between patients with and without PTR (Table 2). However, in univariate analysis, PTR occurred predominantly in patients with M4 French‐American‐British (FAB) subtype (*P* = .001), rearrangements of *RUNX1‐RUNX1T1* (*P* = .026) or *CBFB‐MYH11* (*P* < .001), and c‐*Kit* mutation (*P* = .047), but not inclined to manifest in patients with *NPM1*, *CEBPA*, and *FLT3*‐ITD mutations (Table 2). Notably, rearrangements of *RUNX1‐RUNX1T1* (OR = 5.025, [1.762‐14.329], *P* = .003) or *CBFB‐MYH11* (OR = 20.285, [4.121‐99.848], *P* < .001), which were commonly referred to as core binding factor AML (CBF‐AML), were independently associated with the occurrence of PTR in multivariate analysis (Table 3).

**Table 2:** Clinical features of PTR in patients with de novo AML

	PTR	Non‐PTR	*P*‐value
Total, n (%)	66 (11.8)	494 (88.2)	
WBC(×10^9^/L), median (IQR)	11.4 (3.42‐47.20)	10.60 (2.83‐40.15)	.902
PLT(×10^9^/L), median (IQR)	31 (22.25‐50.25)	34 (23.0‐54.0)	.141
Bone marrow blasts (%, IQR), median	77.3 (50.25‐94.5)	73.5 (43.5‐87.0)	.445
Chromosome aberration, n (%)			
Normal karyotype	29/66 (43.9)	255/494 (51.6)	.241
*RUNX1‐RUNX1T1*	12/66 (18.2)	46/494 (9.3)	.026
*CBFB‐MYH11*	16/66 (24.2)	18/494 (3.6)	<.001
*PML‐RARA*	0/66 (0)	48/494 (9.7)	—
*MLLT3‐KMT2A*,	0/66 (0)	3/494 (0.6)	—
*BCR‐ABL1*	1/66 (1.5)	10/494 (2.0)	1.000
*MLLT10‐KMT2A*	0/66 (0)	2/494 (0.4%)	—
*MLLT4‐KMT2A*	0/66 (0)	5/494 (1.0%)	—
Gene mutation, n (%)			
*NPM1*	7/32 (21.9)	45/299 (15.1)	.313
*CEBPA* (biallelic)	1/29 (3.4)	19/278 (6.8)	.706
*FLT3*‐ITD	0/29 (0)	52/279 (18.6)	—
*c‐Kit*	4/28 (14.3)	12/278 (4.3)	.047
Morphology, n (%)			
M0	1/66 (1.5)	17/494 (3.4)	.710
M1	7/66 (10.6)	72/494 (14.6)	.384
M2	17/66 (25.8)	113/494 (22.9)	.602
M3	0/66 (0)	48/494 (9.7)	—
M4	14/66 (21.2)	39/494 (7.9)	.001
M5	16/66 (24.2)	137/494 (27.7)	.550
Others^a^, ^a^	11/66 (16.7)	55/494 (11.1)	.190

Abbreviations: AML, acute myeloid leukemia; IQR, interquartile ranges; n, observed number of patients within each treatment group; PTR, platelet transfusion refractoriness.

^a^ Others include M6, M7, AML with multilineage dysplasia and AML without available FAB classification.

**Table 3:** Multivariate analysis of PTR in patients with de novo AML

	Hazard ratio (95% CI)	*P*‐value
Female	1.370 (0.585‐3.206)	.468
Splenomegaly	1.412 (0.535‐3.729)	.486
M4 FAB subtype	2.243 (0.380‐13.231)	.372
*c‐Kit* mutation	1.047 (0.255‐4.305)	.949
*RUNX1‐RUNX1T1*	5.025 (1.762‐14.329)	.003
*CBFB‐MYH11*	20.285 (4.121‐99.848)	<.001

Abbreviation: CI, confidence interval.

### 3.3 PTR frequently manifested in patients with CBF‐AML

**FIGURE 1:**
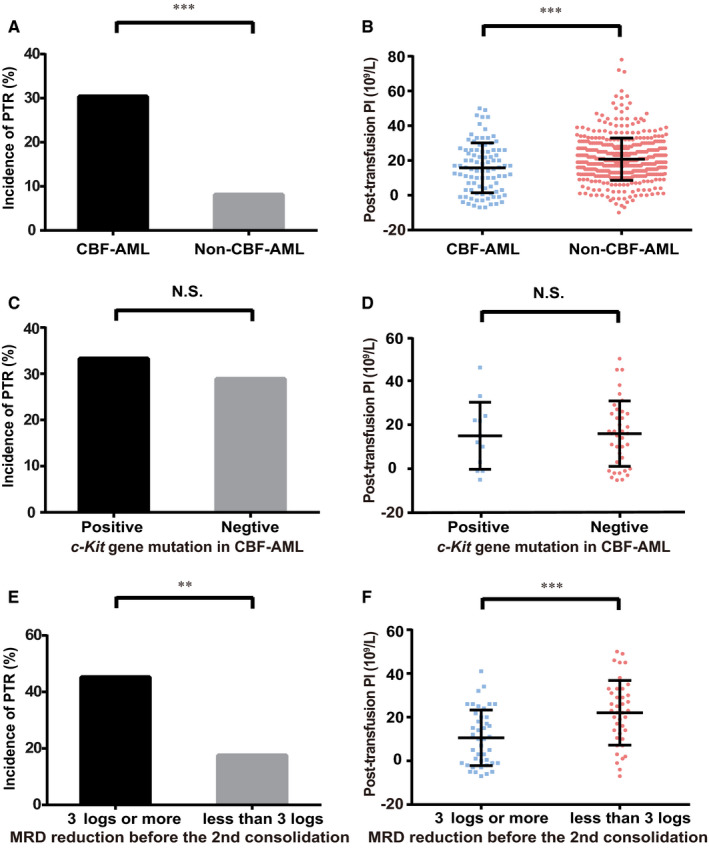
Incidence of PTR and posttransfusion PI in patients with CBF‐AML. Patients with CBF‐AML had higher incidence of PTR (A) and lower posttransfusion PI (B) than patients without. Among the patients with CBF‐AML, the incidence of PTR (C) and posttransfusion PI (D) was comparable in patients with or without *c‐Kit* gene mutation. However, higher incidence of PTR (E) and lower posttransfusion PI (F) revealed in patients who further had better MRD reduction (≥3‐log) before the second consolidation chemotherapy. The midline, bottom, and top lines represent the median value, the 25th and the 75th percentiles, respectively. The statistical differences were analyzed by chi‐square test or unpaired two‐tailed student's *t* test. CBF‐AML: core binding factor acute myeloid leukemia; MRD: minimal residual disease; NS indicates no significant difference; **P  < .010; ***P < .001; PI: platelet count increment; PTR: platelet transfusion refractoriness

PTR manifested in 30.4% (28/92) of the patients who had CBF‐AML, in contrast to 8.1% (38/468) of the others, indicating a strong correlation between PTR and CBF‐AML (*P* < .001, Figure 1A, Figure S2A,B). Thus, when received from random donors, the median posttransfusion PI was more significantly reduced in patients with CBF‐AML than in other patients (*P* < .001, Figure 1B). Among patients with CBF‐AML, the incidence of PTR (33.3% vs 28.9%, *P* = 1.000, Figure 1C) and the posttransfusion PI (*P *= .847, Figure 1D) was comparable in patients with or without *c‐Kit* mutations. Intriguingly, PTR was dramatically developed in patients who achieved 3‐log or more MRD reduction before the second consolidation chemotherapy than in patients who did not achieved it (45.2% vs 17.5%, *P* = .007, Figure 1E). Accordingly, when received from random donors, the median posttransfusion PI was markedly decreased in patients who had better MRD reduction (*P* < .001, Figure 1F).

### 3.4 Outcomes

In CBF‐AML patients, there were 6/92 (6.5%) who died in the first month after the commence of induction therapy, which was defined as an early death (Table S1). Among these patients, 5/28 (17.9%) early death occurred in PTR group compared to 1/64 (1.6%) in non‐PTR group (*P *= .009). And early death secondary to bleeding was obviously higher in patients who had PTR (10.7% vs 0). In CBF‐AML patients, there was no statistical difference in overall survival (OS) between PTR and non‐PTR group (*P *= .745, Table S1, Figure S3A). When early death was excluded in both groups, the OS in PTR group showed a tendency of longer survival, but the difference was still not statistically significant (6‐month, 1‐year, 2‐year, and 5‐year OS rates: 100, 94.1, 72.1, and 46.4% in PTR group vs 93.2, 75.6, 65.9, and 40.0% in non‐PTR group, *P *= .337, Table S1, Figure S3B).

In all PTR patients, 7/66 (10.6%) have an early death, including 5/28 (17.9%) in CBF‐AML group and 2/38 (5.3%) in non‐CBF‐AML group (*P *= .125, Table S2). And early death secondary to bleeding was comparable between CBF and non‐CBF‐AML group (10.7% vs 5.3%, *P *= .643). No difference in OS was observed between PTR patients with CBF and non‐CBF‐AML (Figure S3C, *P* = .294), except patients with early death were excluded (Figure S3D, *P* = .040).

### 3.5 High‐throughput HLA antibody screening

**FIGURE 2:**
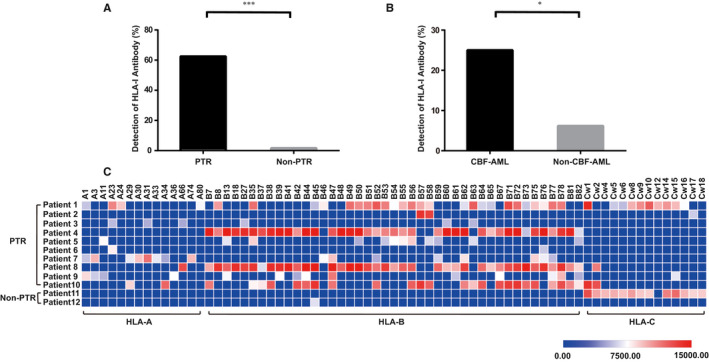
High‐throughput HLA antibody screening in newly diagnosed AML patients. HLA‐I antibodies were detected in 9.0% (12/133) of 133 de novo AML patients. Compared to other patients, antibodies were enriched in patients with PTR (A, 62.5% vs 1.7%, *P* < .001) or in patients with CBF‐AML (B, 25.0% vs 6.2%, *P* = .018). Moreover, a total of 71 HLA serotypes were identified, with 60.56% (43/71) of the identified epitopes belonging to HLA‐B locus. The epitope distribution of the identified HLA‐I antibodies was illustrated in panel C. The color gradient from blue to red represents the increasing value of MFI from 0 to 15 000.00. Positive cutoff: MFI > 5000. CBF‐AML: core binding factor acute myeloid leukemia; HLA‐I: human leukocyte antigen class I; MFI: mean fluorescence intensity; *P < .050; ***P < .001; PTR: platelet transfusion refractoriness

Using Luminex single antigen beads, we examined anti‐HLA‐I antibodies and screened their HLA specificities in the serum from 133 de novo AML patients. HLA‐I antibodies were detected in 9.0% (12/133) among these patients, including in 62.5% (10/16) of patients with PTR and in 1.7% (2/117) of non‐PTR patients (*P* < .001, Figure 2A). Among the 12 patients who had anti‐HLA‐I antibodies, 10 (83.3%) developed PTR. Furthermore, HLA‐I antibodies were detected in 25.0% (5/20) of patients with CBF‐AML, while only in 6.2% (7/113) of the non‐CBF‐AML patients (*P* = .018, Figure 2B).

### 3.6 Epitope HLA‐B closely related to PTR

A total of 71 HLA serotypes were identified (Figure 2C), among which 14 were specific for HLA‐A epitopes, 43 for HLA‐B epitopes, and 14 for HLA‐C epitopes. HLA‐B77 was the most frequently (50.0%, 6/12) identified serotype. HLA‐B35, B45, B47, B52, B56, B62, B75, B76, B78, and B82 were commonly detected as well (41.7%, 5/12) in patients with PTR. However, two HLA‐I antibodies, targeting HLA‐B45 and HLA‐C epitopes, were detected in 2 non‐PTR patients separately (Figure 2C). HLA‐B45, the solely detected serotype in one non‐PTR patient, was identified with marginal MFI intensity (6684.58). HLA‐C epitope, identified in the other non‐PTR patient, had been previously considered to be expressed on the surface of platelet but not contributing to PTR.^26^

## 4 DISCUSSION

PTR is a severe complication with increasing risks of bleeding and mortality in patients who received chemotherapy or hematopoietic stem cell transplantation (HSCT). It had been reported in 4.8% (41/897) of adult AML patients who received intensive chemotherapy, and was more frequently occurred in parous women or in patients with extramedullary disease, leukocytopenia, infection, or hemophagocytic syndrome.^3^ A clinical trial to Reduce Alloimmunization to Platelets (TRAP) conducted in 533 AML patients found that PTR, with an incidence rate of 27%, was associated with positive lymphocytotoxic antibody, heparin administration, fever, bleeding, increasing number of platelet transfusions, increasing weight, at least 2 pregnancies, and male gender. While increasing the dose of platelets transfused or transfusing filtered apheresis platelets could reduce the incidence of PTR.^4^ Among the patients undergoing HSCT, PTR is also a frequent and complex complication. In a study of 167 transplant patients, patients with PTR had lower infusion dose of CD34^+^ cells, higher usage of antibiotics, presence of anti‐HLA‐I antibodies, or reduced‐intensity conditioning regimen. ^10^ In the present study, PTR was more frequently developed in female patients and in patients with splenomegaly, M4 FAB subtype, *c‐Kit* gene mutation, and *RUNX1‐RUNX1T1* or *CBFB‐MYH11* rearrangements.

CBF‐AML, accounting for approximately 15% of adult AML, is defined by the presence of the fusion genes *RUNX1‐RUNX1T1* or *CBFB‐MYH11*. MRD has been used in clinical practice to identify CBF‐AML patients with higher risk of relapse. Several studies have revealed that a 3‐log or more MRD reduction before the second consolidation is a powerful predictor of lower relapse in patients with CBF‐AML.^27^, ^28^, ^29^ In the present study, patients with CBF‐AML had higher risk to develop HLA‐I antibodies and PTR, suggesting increased autoimmune responses to HLA‐I in patients with CBF‐AML.

Upregulated expression of CD80 and CD86 on blast cells was observed in patients with M4 FAB subtype, about half of them carried *CBFB‐MYH11* rearrangement, and was correlated with prolonged remission after induction therapy. The overexpression of these co‐stimulatory molecules on AML blasts may provoke potent autologous cytotoxic T‐cell (CTL) response, which may mediate immune recognition to both platelets and AML blasts because of the widely expressed HLA molecules on their surface.^30^, ^31^, ^32^ Therefore, platelets can be cleared by the activated CTL posttransfusion through antibody‐dependent or ‐independent manner. And the provoked antileukemia effect may reduce the risk of relapse and prolong the duration of remission after chemotherapy. However, the difference in OS rates between PTR and non‐PTR among CBF‐AML patients did not reach the statistical significance. The survival might be complicated by many other factors and more data with bigger sample size may be able to further clarify the relationship between PTR and CBF‐AML.

Moreover, the significant enrichment of HLA‐I antibodies in our patients with PTR is consistent with the notion that alloimmunization to HLA‐I epitopes is the primary cause of immune‐mediated PTR. HLA‐I molecules are expressed as transmembrane glycoproteins on the surface of platelets and all nucleated cells. And HLA molecules can be endocytosed from the plasma, accounting for another source of HLA‐I epitopes on the surface of platelet. Abundant serum antibodies to HLA‐I epitopes react with the transfused platelets and lead to PTR. Therefore, rituximab, romiplostim, plasma exchange, and intravenous immunoglobulins have been widely used in patients with severe HLA alloimmune PTR, even in patients with myeloid malignancies. ^33^, ^34^, ^35^ Moreover, the heavy chain of the HLA‐I molecule is encoded by genes at HLA‐A, HLA‐B, and HLA‐C loci. The most commonly identified epitopes in our patients with PTR belonged to HLA‐B, which suggested that a strategy of “epitope avoidance transfusion” could be used. In this strategy, the actual epitope of HLA antibody should be screened and the transfusion of HLA‐alloimmunized platelets should be avoided. Indeed, improved higher posttransfusion PI had achieved in patients with PTR when transfused with HLA‐matched platelets instead of platelets from random donors.

PTR, an intractable clinical issue in patients with AML, is still full of challenges and demands prompt solutions. We found patients with CBF‐AML had higher risk to develop HLA‐I antibodies and PTR, which depicted novel features of PTR in AML and might provide insights into its efficient managements.

## CONFLICT OF INTEREST

The authors declare that they have no conflict of interests.

## AUTHORS’ CONTRIBUTIONS

JFZ and LH designed and supervised this study; YX, DJL, XLH, HDC, LZ, YL, JZ, YH, ZYD, and HLL enrolled patients, collected and analyzed data; LH and XLH wrote and revised the manuscript. All authors read and approved the final manuscript.

## Supporting information

Fig S1‐S3

Table S1‐S2

Supplementary Material

## Data Availability

The data used to support the findings of this study are available from the corresponding author upon request.
