# Supplementary material for: Clinical and immunological features of platelet transfusion refractoriness in young patients with de novo acute myeloid leukemia
Source: Cancer Med. 2020 May 18;9(14):4941–8. doi: 10.1002/cam4.3140 (PMC7367618; doi:10.1002/cam4.3140)
Supplement: Supplementary file 1 — Fig S1‐S3 [file CAM4-9-4941-s001.docx]

**Supplementary Figure 1. Post-transfusion PI of HLA-matched and non-matched platelets transfusion**


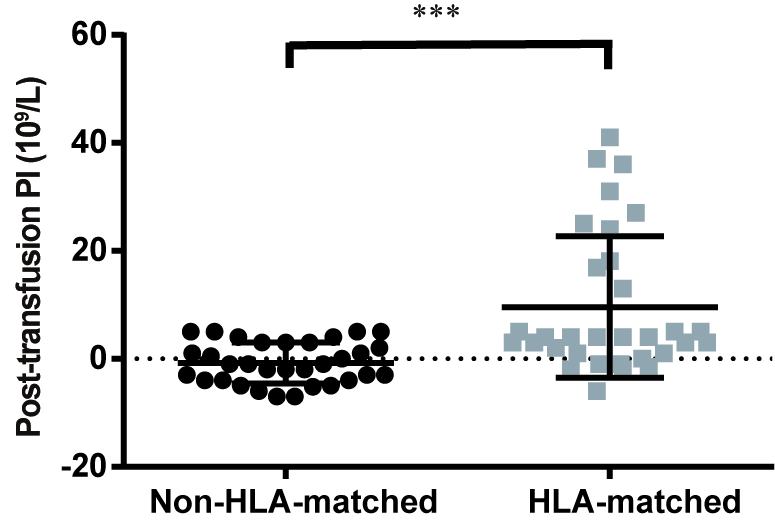


**Supplementary Figure 1. Post-transfusion PI of HLA-matched or not matched platelet transfusion.** The post-transfusion PI of HLA-matched platelets transfusion was significantly higher when compared with non-HLA-matched infusion (*P* < .001). ****P* < .001 compared with the control group.

**Supplementary Figure 2. Incidence of PTR in patients with *RUNX1-RUNX1T1* or *CBFB-MYH11* rearrangements**


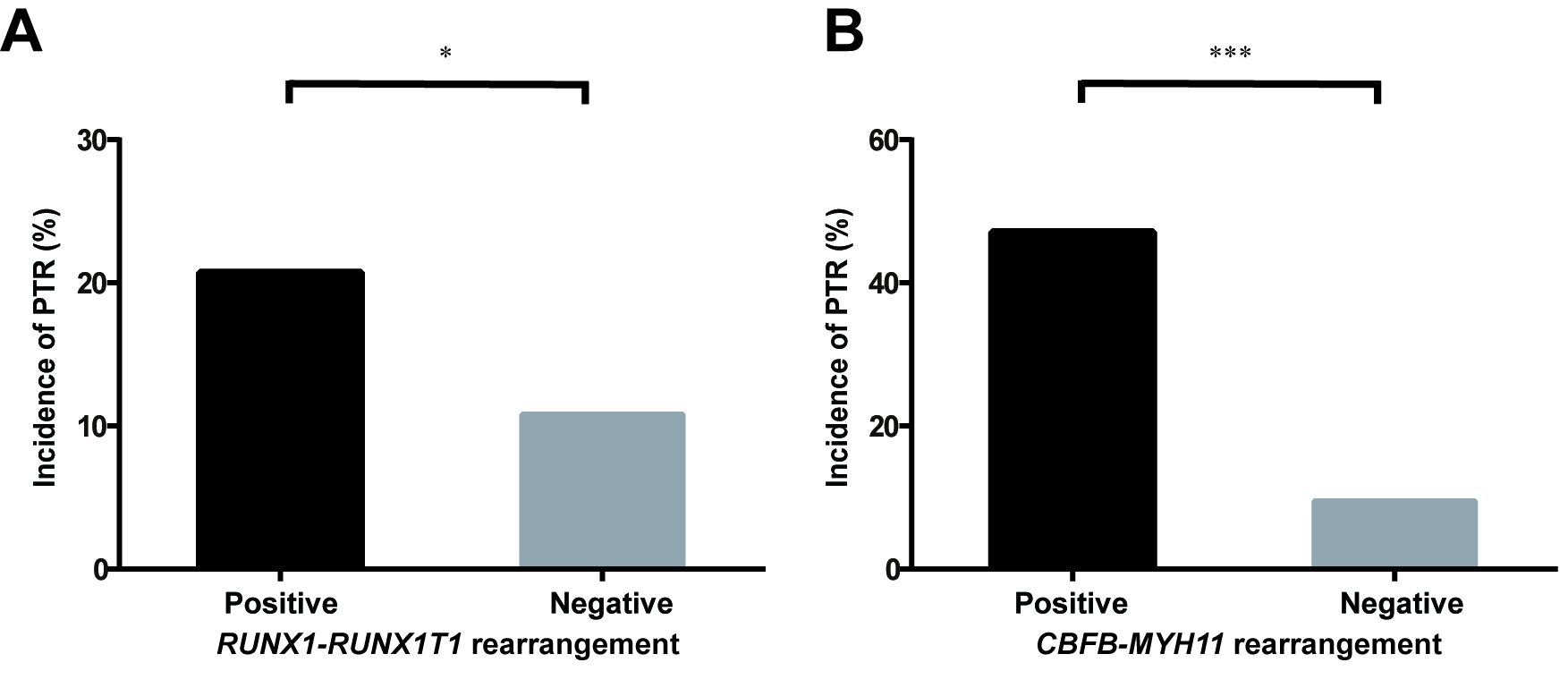


**Supplementary Figure 2****. Incidence of PTR in patients with *RUNX1-RUNX1T1* or *CBFB-MYH11* rearrangements.** Compared to others, patients with *RUNX1-RUNX1T1* (A, *P* = .026) or *CBFB-MYH11* (B, *P* < .001) rearrangements had higher incidence of PTR. The statistical differences were analyzed by chi-square test. **P* < .050 and ****P* < .001 compared with the control group.

**Supplementary Figure 3. Overall survival (OS) in CBF-AML and PTR patients**


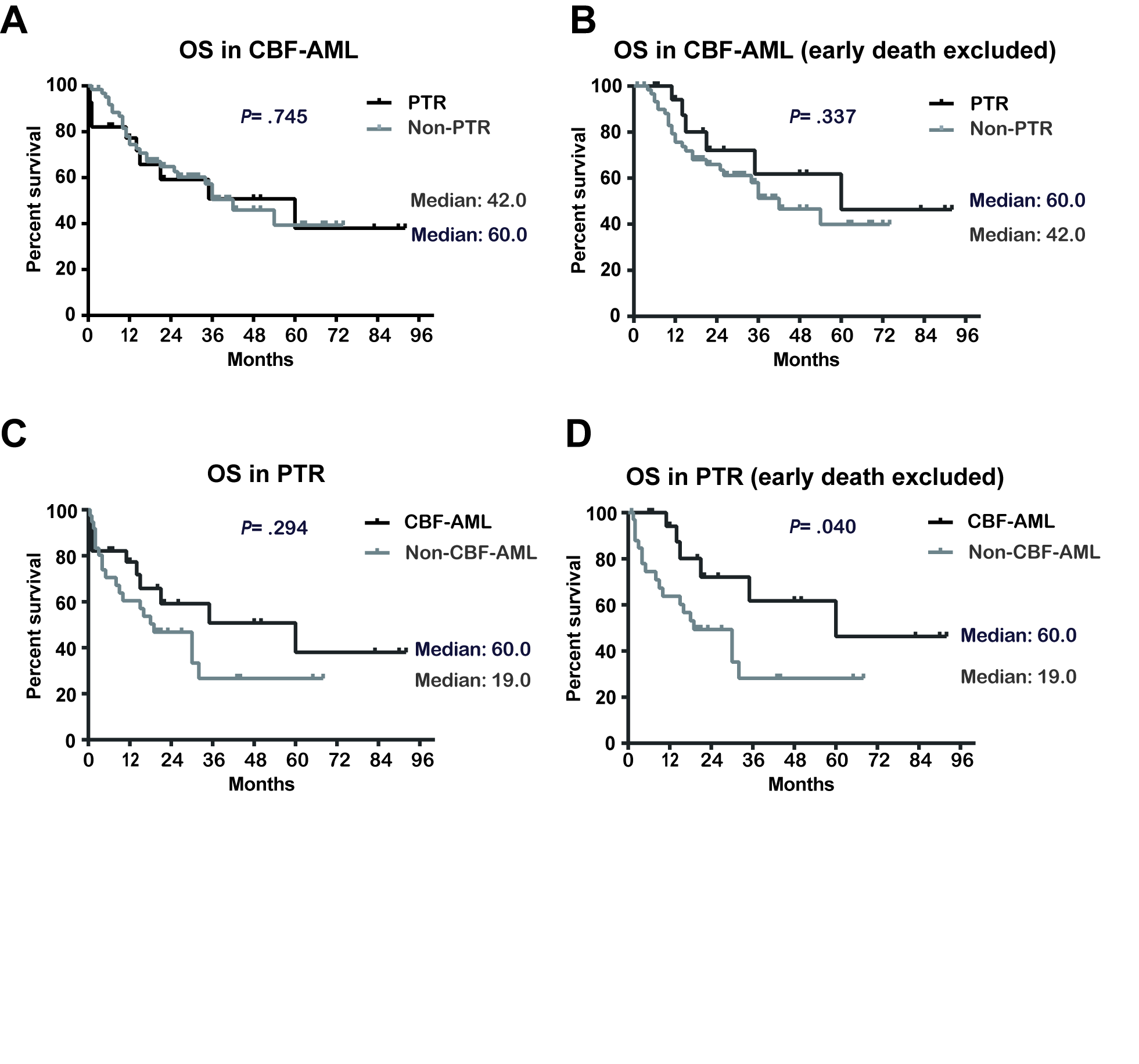


**Supplementary Figure 3. Overall survival (OS) in CBF-AML or PTR patients.** In CBF-AML patients, the OS was comparable between PTR and non-PTR group (A, *P* = .745), even patients with early death was excluded (B, *P* = .337). No difference in OS was observed between PTR patients with CBF and non-CBF-AML (C, *P* = .294), except patients with early death were excluded (D, *P* = .040).
