# Supplementary material for: Clinical and immunological features of platelet transfusion refractoriness in young patients with de novo acute myeloid leukemia
Source: Cancer Med. 2020 May 18;9(14):4941–8. doi: 10.1002/cam4.3140 (PMC7367618; doi:10.1002/cam4.3140)
Supplement: Supplementary file 2 — Table S1‐S2 [file CAM4-9-4941-s002.docx]

**Supplementary Table 1: Outcomes in CBF-AML**

|  | PTR | Non-PTR | *P*-value |
| --- | --- | --- | --- |
| Early death, n (%) | 5/28 (17.9) | 1/64 (1.6) | .009 |
| Early death secondary to bleeding, n (%) | 3/28 (10.7) | 0/64 (0) |  |
| Overall survival |  |  |  |
| Median (months) | 60.0 | 42.0 | .745 |
| 6-month (%) | 82.1 | 95.1 |  |
| 1-year (%) | 77.3 | 74.4 |  |
| 2-year (%) | 59.2 | 64.9 |  |
| 5-year (%) | 38.1 | 39.4 |  |
| Overall survival,  early death excluded |  |  |  |
| Median (months) | 60.0 | 42.0 | .337 |
| 6-month (%) | 100 | 93.2 |  |
| 1-year (%) | 94.1 | 75.6 |  |
| 2-year (%) | 72.1 | 65.9 |  |
| 5-year (%) | 46.4 | 40.0 |  |

**Supplementary Table 2: Outcomes in PTR**

|  | CBF | Non-CBF | *P*-value |
| --- | --- | --- | --- |
| Early death, n(%) | 5/28(17.9) | 2/38(5.3) | .125 |
| Early death secondary to bleeding, n (%) | 3/28(10.7) | 2/38(5.3) | .643 |
| Overall survival |  |  |  |
| Median (months) | 60.0 | 19.00 | .294 |
| 6-month (%) | 82.1 | 70.5 |  |
| 1-year (%) | 77.3 | 60.4 |  |
| 2-year (%) | 59.2 | 46.8 |  |
| 5-year (%) | 38.1 | 26.7 |  |
| Overall survival,  early death excluded |  |  |  |
| Median (months) | 60.0 | 19.00 | .040 |
| 6-month (%) | 100 | 74.4 |  |
| 1-year (%) | 94.1 | 63.8 |  |
| 2-year (%) | 72.1 | 49.4 |  |
| 5-year (%) | 46.4 | 28.2 |  |
