# Supplementary material for: Clinical and immunological features of platelet transfusion refractoriness in young patients with de novo acute myeloid leukemia
Source: Cancer Med. 2020 May 18;9(14):4941–8. doi: 10.1002/cam4.3140 (PMC7367618; doi:10.1002/cam4.3140)
Supplement: Supplementary file 3 — Supplementary Material [file CAM4-9-4941-s003.docx]

**Supplementary Methods**

**Data collection**

From June 2012 to June 2018, a total of 674 cases were collected and 560 cases were eligible for study entry with the following exceptions: 1. patients without chemotherapy or regular platelet counts monitoring; 2. patients with secondary MDS/MPN-transformed AML.

Clinical data consisted of patients’ age, gender, the history of blood transfusion or autoimmune diseases, the existence of fever, infection or splenomegaly, the use of antibiotics and liposomal amphotericin B. Laboratory data included white blood cell (WBC) count, platelet count (PLT), bone marrow blasts count, chromosome aberration, gene mutations (*NPM1*, biallelic *CEBPA*, *FLT3-*ITD, *c-Kit* ) and morphology. Real-time quantitative polymerase chain reaction (RT-PCR) techniques were used for the detection and quantification of the fusion genes to evaluate minimal residual disease (MRD).

Moderate to severe bleeding was defined as deadly bleeding, intracerebral bleeding or substantial hemodynamic compromise requiring treatment, bleeding requiring transfusion according to criteria developed by the Global Use of Strategies to Open Coronary Arteries (GUSTO) study group. (1) Patients with daily body temperature above or equal to 38.4°C was considered to have fever.

**Risk stratification and treatment**

The classification of cytogenetic risks depended on the acknowledged guidelines. AML with favorable outcome included CBF-AML with t(8;21), int(16) or t(16;16) and acute promyelocytic leukemia (APL) with t(15;17); those with adverse prognosis included complex cytogenetics, monosomal karyotype as -5, -7, 5q-, 7q-, 11q23 abnormalities other than t(9;11), abnormal 3q, t(6;9), t(9;22) and those with other genetic aberrations, including a normal karyotype, had an intermediate prognosis.

A standard induction treatment, namely standard-dose cytarabine 100-200 mg/m­­­^2^ continuous infusion for 7 days with idarubicin 10-12 mg/m^2^ or daunorubicin 60 mg/m^2^ for 3 days, were given to patients with non-promyelocytic AML. And patients with acute promyelocytic leukemia (APL) received target agents: all-trans retinoic acid (ATRA) and arsenic trioxide (ATO). (2)

**Supplementary References**

1. Serebruany VL, Atar D. Assessment of Bleeding Events in Clinical Trials—Proposal of a New Classification. The American Journal of Cardiology. 2007;99(2):288-90.

2. McCulloch D, Brown C, Iland H. Retinoic acid and arsenic trioxide in the treatment of acute promyelocytic leukemia: current perspectives. Onco Targets Ther. 2017;10:1585-601.
